# Prevalence of Colonization with Multidrug-Resistant Bacteria: Results of a 5-Year Active Surveillance in Patients Attending a Teaching Hospital

**DOI:** 10.3390/antibiotics12101525

**Published:** 2023-10-10

**Authors:** Angela Quirino, Claudia Cicino, Giuseppe Guido Maria Scarlata, Nadia Marascio, Gianfranco Di Gennaro, Giovanni Matera, Francesca Licata, Aida Bianco

**Affiliations:** 1 Unit of Clinical Microbiology, Department of Health Sciences, University of Catanzaro “Magna Græcia”, 88100 Catanzaro, Italy; quirino@unicz.it (A.Q.); claudiacicino@gmail.com (C.C.); giuseppeguidomaria.scarlata@unicz.it (G.G.M.S.); nmarascio@unicz.it (N.M.); gm4106@gmail.com (G.M.); 2 Department of Health Sciences, School of Medicine, University of Catanzaro “Magna Græcia”, 88100 Catanzaro, Italy; gianfranco.digennaro@unicz.it; 3 Department of Medical and Surgical Sciences, School of Medicine, University of Catanzaro “Magna Græcia”, 88100 Catanzaro, Italy; a.bianco@unicz.it

**Keywords:** antimicrobial resistance, colonization, Gram-negative bacteria, infection control, methicillin-resistant *Staphylococcus aureus*, multidrug-resistant microorganism, surveillance swabs

## Abstract

Combating antimicrobial resistance (AMR) requires comprehensive efforts, such as screening to identify patients colonized by multidrug-resistant microorganisms (MDROs). The primary purpose of this study was to estimate the AMR pattern of methicillin-resistant *Staphylococcus aureus* (MRSA) isolated from nasal surveillance swabs and MDROs isolated from pharyngeal and rectal surveillance swabs in patients attending a teaching hospital. Data were sought retrospectively, from 1 January 2017 to 31 December 2021, from the records produced by the hospital microbiology laboratory. Duplicate isolates, defined as additional isolates of the same microorganism with identical antibiograms, were excluded. Among *Staphylococcus aureus* isolates from nasal swabs, 18.2% were oxacillin-resistant. Among Gram-negative bacteria, 39.8% of *Klebsiella pneumoniae* and 83.5% of *Acinetobacter baumannii* isolates were carbapenem-resistant. Resistance to three antibiotic categories was high among *Acinetobacter baumannii* (85.8%) and *Klebsiella pneumoniae* (42.4%). The present data highlight a high prevalence of MDRO colonization among patients admitted to the hospital and suggest that screening for MDROs could be an important tool for infection control purposes, especially in geographical areas where limiting the spread of MDROs is crucial. The results also underline the importance of active surveillance, especially for carbapenem-resistant, Gram-negative bacteria in reducing their transmission, especially in high-risk units.

## 1. Introduction

Antimicrobial resistance (AMR) has emerged as one of the leading public health threats of the 21st century [1]. Recent data have estimated the magnitude of the AMR burden and the leading pathogen–drug combinations contributing to the AMR threat in different areas [1,2,3]. In Italy, the AMR situation poses a major public health threat to the country. The percentages of resistance to the main classes of antibiotics for the “priority” pathogens remain high and are increasing over time [4]. In particular, the levels of carbapenem-resistant *Enterobacteriaceae* (CRE) and *Acinetobacter baumannii* have now reached hyper-endemic levels, and a constant incidence increase in nosocomial infections by *Klebsiella pneumoniae*, *Staphylococcus aureus*, and *Enterococcus faecium* has occurred over the years starting from 2019 up to 2022 [5,6]. Together with methicillin-resistant *Staphylococcus aureus* (MRSA), this situation has resulted in Italy having one of highest levels of antibiotic resistance among the European Union states [7]. In this context, it is pivotal to understand the leading pathogen–drug combinations contributing to AMR in order to make informed and location-specific policy decisions, particularly about infection prevention and control programs.

If left unchecked, the spread of AMR could make many bacterial pathogens much more lethal in the future than they are today. Infections caused by multidrug-resistant organisms (MDROs) constitute a major challenge in the management of hospitalized patients. Multidrug resistance is the main reason for therapeutic failure, and it is of considerable concern considering that the development of new antibiotic molecules has decreased dramatically over the last ten years [8]. Among the main options for reducing the identified risks of AMR, extensive evidence supports the use of active surveillance cultures for high-risk patients [9,10]. The European Center for Disease Prevention and Control (ECDC) report regarding the effect of infection control measures in preventing the transmission of CRE stated that active rectal screening at the time of admission to a hospital or a specific ward and during an outbreak can effectively limit and prevent the spread of CRE [2,11]. Surveillance swabs can be routinely used as a screening assay to detect MRSA and other MDROs in patients attending selected hospital wards [12]. Moreover, the active surveillance of carriage by MDROs may be an effective tool for the early detection of AMR patterns of pathogens to define the local epidemiology of MDROs [13]. In Italy, the first agency to produce a document for CRE control was the Emilia Romagna Region, in 2011 [14]. One recommendation to healthcare facilities in the region was to start the active surveillance of asymptomatic carriers and take contact isolation precautions for laboratory-confirmed positive cases [14,15].

In Italy, limited data on carriage by MDROs have been published [13,16,17,18]. In a multicenter point-prevalence study, a remarkably high rate of colonization by MDROs was observed among long-term care facility residents. More than 60% of the residents were colonized by at least one MDRO species [17]. With the foregoing considerations in mind, the primary aim of this study was to assess the AMR pattern of MRSA isolated from nasal surveillance swabs and of MDROs isolated from pharyngeal and rectal surveillance swabs over a 5-year period in patients attending a teaching hospital in the Southern part of Italy, considering that these are valuable pieces of information that could aid in the response to the AMR threat. The secondary aims were to analyze trends over time and to compare each other in terms of the proportion of resistance to the main classes of antibiotics.

## 2. Results

Among all hospital admissions, 4109 patients fulfilled at least one eligibility criterion, and 57.7% of them were included in the study (Figure S1, Supplementary Material). A total of 7116 screening swabs, 2372 for each selected body site (i.e., nasal, pharyngeal, and rectal), were collected from 2372 eligible patients; of these, 70% were male. The age of the study population ranged from 18 to 98 years, with a mean of 66.1 (SD ± 17.4). The largest number of swabs were collected by the Cardiac Surgery (*n =* 2694, 37.9%), followed by the Intensive Care Unit (ICU) (*n =* 2358, 33.1%) and the Cardiovascular ICU (CICU) (*n =* 834, 11.7%).

The prevalence of *Staphylococcus aureus* isolates identified through nasal swabs was 20.8%. The distribution of the antimicrobial-resistant phenotype among those isolates showed a resistance rate to benzylpenicillin of 73.9% (Table 1). The resistance to macrolides and lincosamides was approximately 30%. Among those isolates, 18.2% were found to be resistant to oxacillin. The proportion of *Staphylococcus aureus* strains resistant to glycopeptides was 3%. Twenty-five isolates (5.1%) were resistant to at least one agent in three antimicrobial categories. The most frequent combined AMR pattern was penicillins, aminoglycosides, and fluoroquinolones (3%), followed by penicillins, fluoroquinolones, and glycopeptides (2.2%) and penicillins, aminoglycosides, and glycopeptides (1.7%).

The prevalence of Gram-negative microorganisms identified by active surveillance was: 34% *Klebsiella pneumoniae*, 16% *Escherichia coli* and *Enterobacter* spp., 15% *Acinetobacter baumannii*, and 9% *Pseudomonas aeruginosa*. Table 2 displays the prevalence of resistance to the tested antimicrobial agents among the Gram-negative isolates from pharyngeal swabs. The proportion of resistance to carbapenem agents ranged from 25.8% (ertapenem) to 37.1% (imipenem) for *Klebsiella pneumoniae.* Resistance to colistin was detected in 10.3% of *Klebsiella pneumoniae.*

The distribution of antimicrobial-resistant phenotypes among Gram-negative organisms by bacterial species and the tested antimicrobial agents is reported in Table 3. Overall, the resistance pattern to three categories of antimicrobials showed a resistance rate of 18.4% to carbapenems, aminoglycosides, and fluoroquinolones, followed by 14.7% to third- or fourth-generation cephalosporins, aminoglycosides, and fluoroquinolones. Finally, among Gram-negative bacteria isolated from pharyngeal swabs, 3.4% and 1.5% were resistant to third- or fourth-generation cephalosporins, aminoglycosides, fluoroquinolones, and polymyxins and carbapenems, aminoglycosides, fluoroquinolones, and polymyxins, respectively. Among *Klebsiella pneumoniae*, 24.2% were resistant to third- or fourth-generation cephalosporins, aminoglycosides, and fluoroquinolones, and 21.2% were resistant to carbapenems, aminoglycosides, and fluoroquinolones. With regard to *Acinetobacter baumannii*, 80.3% were resistant to carbapenems, aminoglycosides, and fluoroquinolones, and 66.7% were resistant to third- or fourth-generation cephalosporins, aminoglycosides, and fluoroquinolones.

Table 4 shows the distribution of AMR phenotypes among Gram-negative organisms according to bacterial species and antimicrobial agents isolated from rectal swabs. *Klebsiella pneumoniae* isolated from rectal swabs exhibited a resistance to colistin of 9.8%. The AMR phenotype pattern of *Acinetobacter baumannii* displayed a resistance to gentamicin, ciprofloxacin, trimethoprim-sulfamethoxazole, and meropenem higher than 90%.

The AMR pattern to three categories of antimicrobials showed an overall resistance rate among Gram-negative organisms of 58.4% to third- or fourth-generation cephalosporins, aminoglycosides, and fluoroquinolones, followed by 45.8% to carbapenems, aminoglycosides, and fluoroquinolones. Finally, regarding the pattern of resistance to the four categories of antibiotics tested, the resistance rate was 8% to third- or fourth-generation cephalosporins, aminoglycosides, fluoroquinolones, and polymyxins, followed by 6.9% to carbapenems, aminoglycosides, fluoroquinolones, and polymyxins, as shown in Table 5. Globally, MDROs were more than half (62.1%) of the Gram-negative bacteria isolated from rectal swabs. A high proportion (91.3%) of *Acinetobacter baumannii* isolates were resistant to carbapenems, aminoglycosides, and fluoroquinolones, and just two isolates (8.7%) were susceptible to three antimicrobial categories. Among *Klebsiella pneumoniae* isolates, 41.3% were resistant to carbapenems, aminoglycosides, and fluoroquinolones, and 62% were resistant to third- or fourth-generation cephalosporins, aminoglycosides, and fluoroquinolones. Overall, more than half (62%) of *Klebsiella pneumoniae* were MDROs.

The results of the equality test for proportions of an AMR pattern of isolates from the pharyngeal swabs showed that, when compared with *Klebsiella pneumoniae*, *Acinetobacter baumannii* demonstrated a significantly higher resistance to the carbapenem agents. In particular, the resistance of *Acinetobacter baumannii* to imipenem was more than double that of *Klebsiella pneumoniae* (73.3% vs. 37.1%; *p* = 0.02). A similar pattern of resistance was shown for meropenem (82% vs. 31.3%; *p* < 0.001) and ertapenem (100% vs. 25.8%; *p* < 0.001). *Acinetobacter baumannii* also showed a significantly higher resistance (*p* < 0.001) to ciprofloxacin (82%) compared with *Klebsiella pneumoniae* (35.4%) and *Escherichia coli* (21%). A statistically significant difference (*p* = 0.03) in the resistance rate to polymyxins between *Klebsiella pneumoniae* (10.3%) and *Escherichia coli* (1.5%) was also shown. The analysis of data from rectal swabs revealed no statistically significant difference in the proportion of ciprofloxacin resistance between *Acinetobacter baumannii* and *Klebsiella pneumoniae* (91% vs. 85%; *p* = 0.46), as well as in the proportion of imipenem resistance (100% vs. 66%; *p* = 0.16). *Escherichia coli* was omitted from the comparison due to insufficient data.

The results of the multilevel logistic regression analysis showed that the odds of an AMR pattern of isolates from pharyngeal swabs were higher in ICU and CICU (OR: 1.74; 95% CI: 1.31–2.33) compared with those in Cardiac Surgery (Table S1, Supplementary Material). No significant differences were found between the odds of an AMR pattern of isolates from nasal and rectal swabs and the independent covariates tested.

Table 6 shows the sentinel AMR pattern and multidrug-resistance (MDR) isolates tested by bacterial species during 2017–2021. In the course of the first 4 years of surveillance (2017–2020), almost one-fourth of *Klebsiella pneumoniae* isolates were carbapenem-resistant (prevalence ranged from 25.6% to 39%), and during 2021, the rate of resistance increased to 52.9%. The combined resistance to at least three antimicrobial categories ranged from 31.7% in 2017 to 54.8% in 2019. Furthermore, after a decreasing trend between 2017 (88.2%) and 2019 (81.8%), a growth of the proportion of carbapenem-resistant *Acinetobacter baumannii* was recorded in 2020 (89.5%) and 2021 (96%). Combined resistance to at least three antimicrobial categories of *Acinetobacter baumannii* had an increasing trend over the period considered, ranging from 64.7% in 2017 to 96% in 2021. Between 2017 and 2021, no significant difference in the yearly proportion of AMR for any of the isolates was displayed by a chi-square analysis. Regarding resistance to carbapenem among *Klebsiella pneumoniae,* it was approximately doubled between 2020 and 2021. Similarly, *Acinetobacter baumannii* showed an increasing trend in resistance to at least one antimicrobial agent in at least three antimicrobial categories.

## 3. Discussion

To the best of the authors’ knowledge, this paper is the first to study the prevalence of MDRO colonization in acute care hospitals in Italy. Previous studies investigated colonization by MDROs in long-term care facility residents [16,17,19]. It seems obvious to state that the implementation of active screening is laborious for the hospital system. Although a surveillance culture represents a meaningful tool for predicting the development of infection and improving antimicrobial stewardship (AMS), it generates a great workload for laboratory personnel and involves considerable expenditures. Indeed, our institution decided to introduce the protocol for MDROs’ active surveillance after an outbreak caused by carbapenem-resistant *Acinetobacter baumannii* in the ICU [20]. To reduce the burden of MDROs, the hospital infection control committee also established appropriate clinical practices that should have been incorporated into routine patient care, such as a group of bundled evidence-based clinical practices for reducing rates of central-venous-line-associated bloodstream infections and ventilator-associated pneumonia. Moreover, emphasis on handwashing was added, as well as on the preemptive use of contact precautions upon admission until culture negativity was proven and the isolation of infected patients.

Previously published data regarding healthcare-associated infections (HAIs) in our teaching hospital showed that MDR phenotypes among Gram-negative isolates were of concern (i.e., 100% of *Pseudomonas aeruginosa*, 91.6% of *Acinetobacter baumannii*, 52.3% of *Klebsiella pneumoniae*, and 40% of *Escherichia coli*) [21]. A recent study evaluating the impact of the COronaVIrus Disease-19 (COVID-19) pandemic on AMR trends in the ICU underlined a high rate of infections with MDROs (88%) isolated from blood, respiratory, and urine cultures [22]. Moreover, two different studies conducted at different times (2011–2014 and 2015–2019, respectively) evaluated the AMR pattern of ESKAPE bacteria from different biological samples and showed increasing resistance to methicillin for *Staphylococcus aureus* (up to 23% in the last five years) and to colistin for *Acinetobacter baumannii* (from 0% to 2% in the last five years) [4,23]. Although not precisely comparable with biological samples isolated from different body site infections, the data from the present surveillance underline that it is more urgent than ever to prioritize efforts towards AMR containment and improve the detection and rapid response to emerging AMR. It is well known that the COVID-19 pandemic has fueled the AMR global crisis due to the increase in the use of antibiotics to treat COVID-19 patients, disruptions to infection prevention and control practices in overwhelmed health systems, and the deviation of resources away from monitoring and responding to AMR threats [24,25]. Appropriate prescription and the optimized use of antimicrobials according to the principles of AMS, as well as the quality of diagnosis and aggressive infection control measures, are strongly needed to reduce the occurrence of MDROs [26,27]. Moreover, the results confirmed the emergence and spread of *Acinetobacter* spp. resistant to most of the available antimicrobial agents. Its ability to survive in a hospital milieu and to persist for extended periods on surfaces makes it a frequent cause of HAIs, and it has led to multiple outbreaks [28,29,30]. To decrease the emergence of resistance in *Acinetobacter* spp., hand hygiene and barrier techniques are important to keeping the spread of infection in check, as well as the implementation and monitoring of AMS programs in hospitals. The high rate of carbapenem resistance (>80%) and MDR among *Acinetobacter baumannii* could also be linked to the inappropriate use of antibiotics, as previously reported [31]. In the authors’ opinion, it is critical to avoid the use of antibiotics in all the conditions where they are not recommended [32,33,34,35,36].

Regarding the prevalence of colonization with MDROs, surveillance data showed that it has progressively increased over the last decade [37], and the present comparison of AMR pattern data over 5 years, although not statistically significant, confirms this trend. In the present study, through an active surveillance culture, MRSA was detected in 89 out of 489 isolates. A recent worldwide systematic review and meta-analysis on the prevalence of MRSA in elderly care centers showed a global prevalence of 14.7%, which, in Italy, increased to 16.3% [38]. In hospital settings, MRSA prevalence ranged between 2.7% [39] and 9.9% among colonized patients [40]. The prevalence of MRSA in our setting is of concern and deserves attention and the implementation of control measures. A hospital-based observational follow-up study exhibited a decrease in the MRSA nosocomial transmission rate after the implementation of a rigorous policy of active surveillance cultures for patients at risk, followed by the strict isolation and eradication of known MRSA carriers [41]. The resistance rate of *Klebsiella pneumoniae* to different carbapenem agents was alarming, ranging from 25.8% (ertapenem) to 37.1% (imipenem). A similar pattern was found in the surveillance of laboratory-confirmed bloodstream infections conducted using data from three diagnostic laboratories in the Calabria region [42]. In Italy, the rate of carbapenem resistance in *Klebsiella pneumoniae* increased very rapidly during the last years, from 1.3% in 2009 to 15% in 2010, 27% in 2011, and 34% in 2013 [43]. This trend was confirmed by the latest ECDC report that showed an increase in resistance rates for *Klebsiella pneumoniae* (up to 55%) from 2016 to 2020 [37]. A slight decrease in the rate of carbapenem resistance was observed in 2017, with percentages just under 30% [44].

The present data highlight an alarmingly high prevalence of MDRO colonization among patients admitted to the hospital and suggest that screening for MDROs could be an attractive strategy for infection control purposes, especially in local areas where limiting the spread of MDROs is crucial, such as Italian regions. Active surveillance is pivotal for the timely detection and separation of carriers to reduce the time during which such unrecognized reservoirs might disseminate MDROs, the activation of contact precautions, and, after careful risk evaluation, antibiotic treatment guidance on suspicion of infection. In hospitals, active surveillance has to be considered, since they have been a major source of the spread of epidemics. Laboratory capacity is the main reason why screening programs of sentinel MDROs could not be performed. The lack of data about the local epidemiology of these bacteria makes decision-making on the choice of the most appropriate empirical antimicrobial therapy more complicated, and it contributes to the use of more broad-spectrum antibiotics for empirical treatment and, ultimately, to the escalation of AMR.

Our study has some limitations. First, our results might overestimate the prevalence rates of MDROs, as our study population consisted of high-risk patients who, in some cases, had been hospitalized at healthcare facilities. Second, the study design (i.e., 5-year active surveillance laboratory-based) did not allow for investigating risk factors for MDRO acquisition, such as previous therapy with antibiotics; thus, potential correlations among selected variables and colonization by MDROs at admission were not analyzed in the present study. However, laboratory-based data without linkage to patient information are frequently used to monitor AMR [45]. Third, a control group was not included in this study since the data were collected by the hospital microbiology laboratory as part of an intervention established to prevent and control MDR infections. However, routinely collected data are increasingly used for biomedical research [46], and the analysis of the data could inform useful descriptive features. Fourth, some sets of swabs were not taken for the eligible patient, and a higher number of male than female patients were included in the study (70% of the sample). This is a real-world study that describes a real epidemiological trend claiming that male individuals are admitted to high-risk units, such as the ICU and the Cardiac Surgery unit, more frequently than females [47,48,49]. Similarly, patients being excluded due to missing samples, during the first wave of the COVID-19 pandemic when surveillance was partially disrupted, is obviously a matter of concern that may introduce a bias. However, the fact that real-world data research is more susceptible to bias compared with conventional studies is well-known. Although real-world data do not always follow the same methods used in studies involving primary data collection, they provide valuable information for assessing new programs and treatments. The main merit of the present real-world data research is helping to understand the burden of AMR and the highest-priority pathogens. In addition, real-world data may identify geographic variation and temporal change in the prevalence of one of the leading public health threats. Fifth, the study was a single-center investigation and, therefore, future large, multi-center studies are needed to confirm the present findings. However, the setting is a teaching hospital which covers the health needs of individuals of the southern part of Italy, and the data may provide a snapshot of the highest-priority MDROs, which is useful in identifying geographic variation and temporal change in the prevalence of MDROs. This information is valuable for resource planning.

## 4. Materials and Methods

### 4.1. Study Design and Patient Population

Data were sought retrospectively from the records produced by the hospital microbiology laboratory of a teaching hospital, from 1 January 2017 to 31 December 2021. The setting is a tertiary care hospital, located in the Calabria Region of Italy, which has 225 beds and covers the health needs of the 341,000 inhabitants of the Catanzaro province (15,000 km^2^) and those of some 1.8 million inhabitants of the Region and 13.5 million inhabitants of the southern part of Italy [50,51]. The data were collected in real time by FREQUENZA v12.5.3, available in METAFORA software (https://www.metafora-biosystems.com/), and stored and updated in a password-protected Excel^®^ spreadsheet. Two authors were independently involved in checking for errors or inconsistencies in the data. According to national guidelines [52] and previously published studies [18,40,53], the hospital committee established the following inclusion criteria for the routine screening at admission of all patients with an age > 18 years who were admitted to high-risk units (i.e., ICU, CICU, and Cardiac Surgery) or had admission in another hospital in the previous 6 months and patients coming from nursing homes for the elderly. Duplicate isolates, defined as additional isolates of the same microorganism with identical antibiograms, were excluded. The patients were screened in the hospital or eligible wards upon admission in accordance with the institutional protocol for the active surveillance of MRSA and MDRO strains by taking a set of nasal, pharyngeal, and rectal swabs. In accordance with the standardized classification of the joined expert panel of the ECDC and the US Centers for Disease Control and Prevention (CDC), isolates that were non-susceptible to at least one agent in ≥three antimicrobial categories were classified as MDRO [54]. Patients may have had more than one type of microorganism, and the data for these patients were counted separately for each microorganism isolation.

### 4.2. Culture Conditions, Identification, and Antimicrobial Susceptibility Testing

According to clinical laboratory guidelines, nasal swabs were cultured using Mannitol Salt 2 Agar (MSA2) (bioMérieux, Grassina, Italy), and pharyngeal and rectal swabs were cultured using chromID^®^ Extended Spectrum ß-Lactamase-producing (ESBL) Enterobacteriaceae (bioMérieux, Italy) and MacConkey Agar (bioMérieux, Italy) at 37 °C in aerobic conditions for 16–18 h. The identification of the isolated bacteria was carried out by a Vitek^®^2 System (bioMérieux, Italy) and matrix-assisted laser-desorption ionization time-of-flight mass spectrometry (MALDI-TOF). Then, antimicrobial susceptibility tests (AST) were performed using a Vitek^®^2 System (bioMérieux, Italy) and Sensititre System (ThermoFisher Scientific, Waltham, MA, USA), according to the European Committee on Antimicrobial Susceptibility Testing (EUCAST) guidelines. Each panel was standardized for Gram-positive and Gram-negative AST profiles comprising the list below:

*Aminoglycoside*: Amikacin (AMK), Gentamicin (GEN); *Cephalosporins*: Cefepime (FEP), Cefoxitin (FOX), Ceftazidime (CAZ), Cefuroxime and Cefuroxime axetil (CXM); *Quinolones*: Ciprofloxacin (CIP), Levofloxacin (LVX); *Penicillin*: Amoxicillin/Clavulanic acid (AMC), Ampicillin (AMP), Oxacillin (OXA), Piperacillin/Tazobactam (TZP); *Carbapenems*: Ertapenem (ETP), Imipenem (IMP), Meropenem (MEM); *Glycopeptides*: Teicoplanin (TEC), Vancomycin (VAN); *Macrolide*: Erythromycin (ERY); *Lincosamides*: Clindamycin (CLI); *Oxazolidinone*: Linezolid (LZD); *Tetracycline*: Tetracycline (TET); *Sulfonamides*: Sulfamethoxazole/Trimethoprim (SXT); *Glycylcyclines*: Tigecycline (TGC); *Polypeptide*: Colistin (CS); *Lipopeptides*: Daptomycin (DAP).

### 4.3. Statistical Analysis

All personal data were anonymized and kept in strict accordance with the patients’ privacy, with just their gender, age, hospital ward, and AST results collected. Continuous variables were described by means and standard deviations if normally distributed or by medians and interquartile ranges in cases of skewness. The global range was also reported. The Shapiro–Wilk test was used to examine the distributions’ shape. Categorical variables were described by percentage counts. In addition, the resistance rates of *Klebsiella pneumoniae*, *Escherichia coli*, and *Acinetobacter baumannii* to carbapenems, fluoroquinolones, and polymyxins in rectal and pharyngeal swabs were compared with each other by an equality test for proportions. *Escherichia coli* isolates from both pharyngeal and rectal swabs were not included in the comparison due to insufficient observations, and the same happened for the comparison between MRSA and MDR *Staphylococcus aureus* isolated from nasal swabs. The 5 × 2 chi-squared tests were conducted to explore the differences in the proportion of the AMR pattern during the 2017–2021 period. Finally, multilevel logistic regression models were built to estimate the association of the sex (0 = female, 1 = male); age (continuous, in years); and hospital ward (Cardiac Surgery = 0, ICU and CICU = 1, other wards = 2) with an AMR pattern of isolates from nasal (Model 1), pharyngeal (Model 2), and rectal swabs (Model 3). Stata Statistical Software, Version 17 (StataCorp LLC: College Station, TX, USA) [55] was used to analyze the data.

### 4.4. Ethical Considerations

The present retrospective study is based on clinical isolates stored in an anonymous archive without association with clinical data. For these reasons, ethical approval and consent to participate are not applicable.

## 5. Conclusions

In conclusion, the study findings underline the potential role of active surveillance, especially testing for carbapenem-resistant, Gram-negative bacteria, which is useful in reducing hospital horizontal transmission, especially in high-risk units, such as the ICU. This approach can facilitate the proper establishment of AMS and infection control measures, preventing, at the same time, the emergence of new MDR strains.

## Figures and Tables

**Table 1:** Distribution of isolates tested (*n*) and percentage of isolates with an antimicrobial-resistant phenotype (% Res) to antimicrobial agents among *Staphylococcus aureus* isolated from nasal swabs.

Antimicrobial Category	Antimicrobial Agents	*Staphylococcus aureus* (Total Isolates = 493)	
		*n*	% Res
Penicillins	PEN	491	73.9
	OXA	489	18.2
Fluoroquinolones	LVX	364	21.2
Aminoglycosides	GEN	363	5.8
Macrolides	ERY	363	32
Lincosamides	CLI	361	29.9
Tetracyclines	TET	363	4.4
Folate pathway inhibitors	SXT	363	2.8
Glycopeptides	VAN	364	0.3
	TEC	362	2.8
Oxazolidinones	LZD	364	0.5
Lipopeptides	DAP	363	0.3

Abbreviations: Res, isolates with an antimicrobial-resistant phenotype; CLI, clindamycin; DAP, daptomycin.; ERY, erythromycin; GEN, gentamicin; LVX, levofloxacin; LZD, linezolid; OXA, oxacillin; PEN, benzylpenicillin; SXT, trimethoprim-sulfamethoxazole; TEC, teicoplanin; TET, tetracycline; VAN, vancomycin.

**Table 2:** Distribution of isolates tested (*n*) and percentage of isolates with an antimicrobial-resistant phenotype (% Res) among Gram-negative organisms by bacterial species and tested antimicrobial agents from pharyngeal swabs.

Antimicrobial Category	Antimicrobial Agent	*Klebsiella* *pneumoniae*		*Escherichia coli*		*Acinetobacter* *baumannii*		*Enterobacter* spp.		*Pseudomonas* *aeruginosa*		Other Gram-Negative Organisms ^1^		Total	
		(Total Isolates = 99)		(Total Isolates = 81)		(Total Isolates = 62)		(Total Isolates = 48)		(Total Isolates = 46)		(Total Isolates = 86)		(Total Isolates = 422)	
		*n*	% Res	*n*	% Res	*n*	% Res	*n*	% Res	*n*	% Res	*n*	% Res	*n*	% Res
Penicillins	AMP	39	100	24	41.7	15	100	NT	-	15	100	8	87.5	86	85.1
Penicillins + β-lactamase inhibitors	AMC	99	38.4	81	35.8	9	100	48	100	9	100	63	66.7	175	56.6
	TZP	98	36.7	81	4.9	NT	-	48	10.4	45	15.6	67	10.4	59	17.4
Second-generation cephalosporins	CXM	11	9.1	14	14.3	NT	-	7	42.9	NT	-	18	22.2	10	20
	FOX	50	16	32	0	19	100	23	100	NT	-	34	41.2	64	40.5
Third- or fourth-generation cephalosporins	CAZ	99	36.4	80	12.5	NT	-	48	12.5	46	13	70	14.3	68	19.8
	FEP	99	35.4	80	10	NT	-	48	4.2	46	13	66	1.5	52	15.3
Aminoglycosides	AMK	97	16.5	81	0	42	73.8	48	0	46	6.5	66	6.1	54	14.2
	GEN	99	17.2	81	11.1	61	82	48	2.1	37	13.5	68	5.9	86	21.8
Fluoroquinolones	CIP	99	35.4	81	21	61	82	48	2.1	46	17.4	70	12.9	120	29.6
Carbapenems	IMP	35	37.1	10	0	15	73.3	12	0	9	0	7	28.6	26	29.5
	MEM	99	31.3	81	0	61	82	48	0	46	10.9	70	7.1	91	22.5
	ETP	89	25.8	81	0	15	100	47	2.1	15	100	65	6.2	58	18.6
Glycylcyclines	TGC	79	5.1	67	0	NT	-	29	3.4	13	100	43	11.6	23	10
Folate pathway inhibitors	SXT	99	25.3	80	25	61	80.3	47	2.1	20	100	81	9.9	123	31.7
Polymyxins	CS	87	10.3	67	1.5	59	0	40	0	38	0	44	47.7	32	9.6

Abbreviations: Res, isolates with an antimicrobial-resistant phenotype; AMC, amoxicillin-clavulanic; AMK, amikacin; AMP, ampicillin, CAZ, ceftazidime; CIP, ciprofloxacin; CS, colistin; SXT, trimethoprim-sulfamethoxazole; FEP, cefepime; CXM, cefuroxime and cefuroxime axetil; ETP, ertapenem; FOX, cefoxitin; GEN, gentamicin; IMP, imipenem; MEM, meropenem; TZP, piperacillin/tazobactam; TGC, tigecycline; NT: not tested. ^1^ *Achromobacter denitrificans*, *Achromobacter xylosoxidans*, *Aeromonas hydrophila/caviae*, *Aeromonas sobria*, *Alcaligenes faecalis*, *Citrobacter* spp. (*C. braakii*, *C. farmer*, *C. freundii*, *C. koseri*, *C. youngae*), *Hafnia alvei*, *Morganella morganii* ssp. *morganii*, *Morganella morganii* ssp. *sibonii*, *Pantoea agglomerans*, *Proteus mirabilis*, *Proteus species*, *Providencia rettgeri*, *Pseudomonas alcaligenes*, *Pseudomonas luteola*, *Pseudomonas putida*, *Raoultella ornithinolytica*, *Raoultella planticola*, *Serratia liquefaciens group*, *Serratia marcescens*, *Serratia odorifera*, *Stenotrophomonas maltophilia.*

**Table 3:** Distribution of isolates tested (*n*) and percentage of isolates with an AMR pattern (% Res) among Gram-negative organisms by bacterial species and tested antimicrobial categories from pharyngeal swabs.

AMR Pattern ^1^	*Klebsiella* *pneumoniae*		*Escherichia coli*		*Acinetobacter* *baumannii*		*Enterobacter* spp.		*Pseudomonas* *aeruginosa*		Other Gram-Negative Organisms ^2^		Total	
	(Total Isolates = 99)		(Total Isolates = 81)		(Total Isolates = 62)		(Total Isolates = 48)		(Total Isolates = 46)		(Total Isolates = 86)		(Total Isolates = 422)	
	*n*	% Res	*n*	% Res	*n*	% Res	*n*	% Res	*n*	% Res	*n*	% Res	*n*	% Res
Carbapenems + aminoglycosides + fluoroquinolones	99	21.2	81	0	61	80.3	61	0	46	8.7	68	0	403	18.4
Third- or fourth-generation cephalosporins + aminoglycosides + fluoroquinolones	99	24.2	81	7.4	18	66.7	18	2.1	46	8.7	68	8.8	360	14.7
Carbapenems + aminoglycosides + fluoroquinolones + polymyxins	87	5.7	67	0	58	0	58	0	38	0	44	0	334	1.5
Third- or fourth-generation cephalosporins + aminoglycosides + fluoroquinolones + polymyxins	87	5.7	67	0	17	0	17	0	38	0	44	11.4	293	3.4

Abbreviations: Res, isolates with resistance to considered antimicrobial categories. ^1^ Resistance was defined as non-susceptibility to ≥one agent in the considered antimicrobial categories. ^2^ *Achromobacter denitrificans*, *Achromobacter xylosoxidans*, *Aeromonas hydrophila/caviae*, *Aeromonas sobria*, *Alcaligenes faecalis*, *Citrobacter* spp. *(C. braakii*, *C. farmer*, *C. freundii*, *C. koseri*, *C. youngae*), *Hafnia alvei*, *Morganella morganii* ssp. *morganii*, *Morganella morganii* ssp. *sibonii*, *Pantoea agglomerans*, *Proteus mirabilis*, *Proteus species*, *Providencia rettgeri*, *Pseudomonas alcaligenes*, *Pseudomonas luteola*, *Pseudomonas putida*, *Raoultella ornithinolytica*, *Raoultella planticola*, *Serratia liquefaciens group*, *Serratia marcescens*, *Serratia odorifera*, *Stenotrophomonas maltophilia.*

**Table 4:** Distribution of isolates tested (*n*) and percentage of isolates with an antimicrobial-resistant phenotype (% Res) among Gram-negative organisms according to bacterial species and antimicrobial agents from rectal swabs.

Antimicrobial Category	Antimicrobial Agent	*Klebsiella* *pneumoniae*		*Acinetobacter* *baumannii*		Other Gram-Negative Organisms ^1^		Total	
		(Total Isolates = 92)		(Total Isolates = 23)		(Total Isolates = 17)		(Total Isolates = 132)	
		*n*	% Res	*n*	% Res	*n*	% Res	*n*	% Res
Penicillins	AMP	17	100	4	100	2	100	23	100
Penicillins + β-lactamase inhibitors	AMC	92	87	3	100	14	92.9	109	88.1
	TZP	92	71.7	NT	-	15	53.3	107	69.2
Second-generation cephalosporins	CXM	1	100	NT	-	NT	-	1	100
	FOX	28	46.4	5	100	2	100	35	57.1
Third- or fourth-generation cephalosporins	CAZ	92	90.2	NT	-	16	87.5	108	89.8
	FEP	92	87	NT	-	16	50	108	81.5
Aminoglycosides	AMK	92	25	18	83.3	16	6.3	126	31
	GEN	92	51.1	23	91.3	15	33.3	130	56.2
Fluoroquinolones	CIP	92	84.8	23	91.3	17	52.9	132	81.8
Carbapenems	IMP	29	65.5	4	100	3	33.3	36	66.7
	MEM	92	48.9	23	91.3	16	93.8	131	47.3
	ETP	75	46.7	4	100	14	21.4	93	45.2
Glycylcyclines	TGC	76	15.8	NT	-	15	6.7	91	14.3
Folate pathway inhibitors	SXT	92	72.8	23	91.3	14	21.4	129	70.5
Polymyxins	CS	92	9.8	23	0	15	6.7	130	7.7

Abbreviations: Res, isolates with an antimicrobial-resistant phenotype; AMC, amoxicillin-clavulanic acid; AMK, amikacin; AMP, ampicillin; CAZ, ceftazidime; CIP, ciprofloxacin; CS, colistin; CXM, cefuroxime; SXT, trimethoprim-sulfamethoxazole; FEP, cefepime; ETP, ertapenem; FOX, cefoxitin; GEN, gentamicin; IMP, imipenem; MEM, meropenem; TZP, piperacillin/tazobactam; TGC, tigecycline; NT, not tested. ^1^ *Citrobacter freundii*, *Enterobacter aerogenes*, *Enterobacter cloacae complex*, *Escherichia coli*, *Klebsiella oxytoca*, *Pseudomonas aeruginosa.*

**Table 5:** Distribution of isolates tested (*n*) and percentage of isolates with an AMR pattern (% Res) among Gram-negative organisms by bacterial species and tested antimicrobial categories from rectal swabs.

AMR Pattern ^1^	*Klebsiella* *pneumoniae*		*Acinetobacter baumannii*		Other Gram-Negative Organisms ^2^		Total	
	(Total Isolates = 92)		(Total Isolates = 23)		(Total Isolates = 17)		(Total Isolates = 132)	
	*n*	% Res	*n*	% Res	*n*	% Res	*n*	% Res
Carbapenems + aminoglycosides + fluoroquinolones	92	41.3	23	91.3	16	6.3	131	45.8
Third- or fourth-generation cephalosporins + aminoglycosides + fluoroquinolones	92	62	5	100	16	25	113	58.4
Carbapenems + aminoglycosides + fluoroquinolones + polymyxins	92	8.7	23	0	15	6.7	130	6.9
Third- or fourth-generation cephalosporins + aminoglycosides + fluoroquinolones + polymyxins	92	8.7	5	0	15	6.7	112	8

Abbreviations: Res, isolates with resistance to considered antimicrobial categories. ^1^ Resistance was defined as non-susceptibility to ≥one agent in the considered antimicrobial categories. ^2^ *Citrobacter freundii*, *Enterobacter aerogenes*, *Enterobacter cloacae complex*, *Escherichia coli*, *Klebsiella oxytoca*, *Pseudomonas aeruginosa.*

**Table 6:** Trends in the percentage of resistance to sentinel antibiotics and selected antimicrobial categories by bacterial species during the years 2017–2021.

Bacterial Species	AMR Pattern	2017		2018		2019		2020		2021		*p*-Values
		*n*	% Res	*n*	% Res	*n*	% Res	*n*	% Res	*n*	% Res	
*Staphylococcus aureus* (493)	Penicillins	102	17.5	94	20.2	76	17.9	94	18.1	123	16.9	0.42
*Klebsiella. pneumoniae* (191)	Carbapenems	41	39	39	25.6	31	35.5	46	28.7	34	52.9	0.08
	Combined resistance ^1^	41	31.7	39	35.9	31	54.8	46	50	34	41.2	0.23
*Escherichia coli* (88)	Carbapenems	17	0	11	9.1	19	0	20	0	21	0	NA
	Combined resistance ^1^	17	11.8	11	9.1	22	13.6	20	10	21	4.8	0.9
*Acinetobacter baumannii* (85)	Carbapenems	17	88.2	12	83.3	11	81.8	19	89.5	25	96	0.68
	Combined resistance ^1^	17	64.7	12	75	11	81.8	19	89.5	25	96	0.08

Abbreviations: Res, resistance to sentinel antibiotics and to combinations of antimicrobial categories. ^1^ Resistance to at least one antimicrobial agent in at least three of carbapenems, aminoglycosides, fluoroquinolones, and third- or fourth-generation cephalosporins.

## Data Availability

The data presented in this study are available on request from the corresponding author.

## Supplementary Materials

The following supporting information can be downloaded at: https://www.mdpi.com/article/10.3390/antibiotics12101525/s1, Figure S1: Flow-chart of sampling procedures of the study; Table S1: Results of the multilevel logistic regression analysis for estimates of associations of AMR pattern of isolates with explanatory variables.
