# Supplementary material for: Prevalence of Colonization with Multidrug-Resistant Bacteria: Results of a 5-Year Active Surveillance in Patients Attending a Teaching Hospital
Source: Antibiotics (Basel). 2023 Oct 10;12(10):1525. doi: 10.3390/antibiotics12101525 (PMC10604483; doi:10.3390/antibiotics12101525)
Supplement: Supplementary file 1 [file antibiotics-12-01525-s001.zip › antibiotics-2552192-supplementary.pdf]

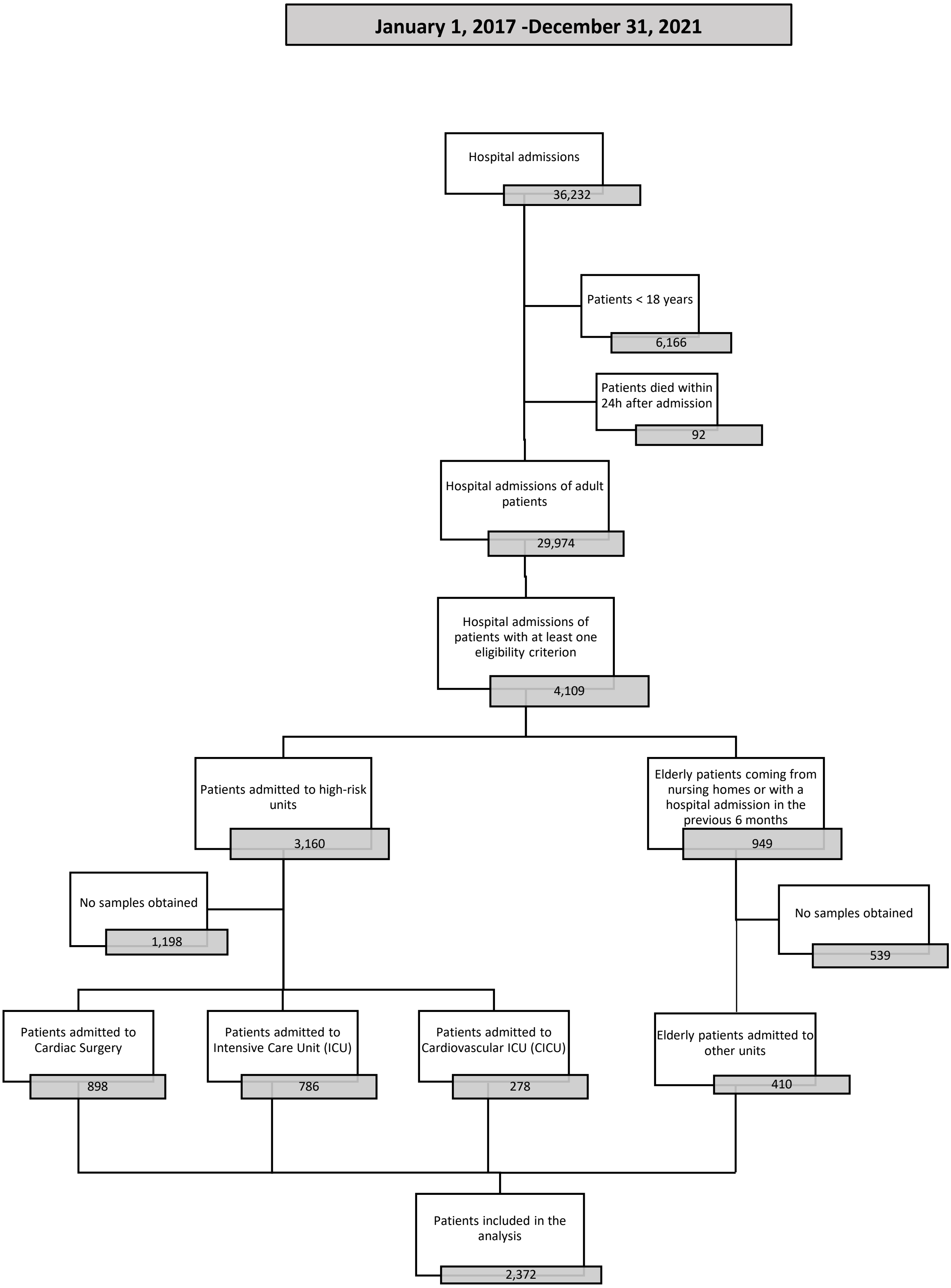

Figure S1. Flow-chart of sampling procedures of the study

Table S1. Results of the multilevel logistic regression analysis for estimates of associations of AMR pattern of isolates with explanatory variables

Model 1: Outcome: *AMR pattern of isolates from nasal swabs*
Log-likelihood =-94924.567; Prob > chi2 = 0.2460; Obs = 493

Variables	OR	95% CI	*p*
Hospital wards			
Cardiac Surgery*	1.00		
ICU and CICU	1.08	0.95-1.22	0.243
Other wards	1.18	0.99-1.41	0.068
Sex			
Female*	1.00		
Male	0.94	0.83-1.06	0.289
Age in years (continuous)	0.99	0.99-1.00	0.932

Model 2: Outcome: *AMR pattern of isolates from pharyngeal swabs*
Log-likelihood =-113078.35; Prob > chi2 < 0.001 ; Obs = 422

Variables	OR	95% CI	*p*
Hospital wards			
Cardiac Surgery*			
ICU and CICU	1.74	1.31-2.33	<0.001
Other wards	0.90	0.64-1.25	0.519
Sex			
Female*	1.00		
Male	1.04	0.78-1.39	0.795
Age in years (continuous)	0.99	0.98-1.00	0.146

Model 3: Outcome: *AMR pattern of isolates from rectal swabs*
Log-likelihood =-30431.049; Prob > chi2 = 0.4123; Obs = 132

Variables	OR	95% CI	*p*
Hospital wards			
Cardiac Surgery*	1.00		
ICU and CICU	1.03	0.80-1.31	0.842
Other wards	0.56	0.26-1.18	0.125
Sex			
Female*	1.00		
Male	0.70	0.41-1.22	0.211
Age in years (continuous)	0.99	0.98-1.01	0.721

Abbreviations: ICU, Intensive Care Unit; CICU, Cardiovascular Intensive Care Unit.
*Reference category.
